# TNFα protein, DNA methylation, mRNA and miRNA expression evaluation in multiple sclerosis

**DOI:** 10.3389/fmolb.2025.1730177

**Published:** 2025-12-17

**Authors:** E. Costantini, N. H. Greig, L. Aielli, P. C. Silva, M. Di Carlo, F. Konstantinidou, M. Reale

**Affiliations:** 1 Department of Innovative Technologies in Medicine and Dentistry, University “G. D’Annunzio”, Chieti, Italy; 2 Drug Design & Development Section, Translational Gerontology Branch, Intramural Research Program, National Institute on Aging, National Institutes of Health, Baltimore, MD, United States; 3 School of Medicine and Biomedical Sciences. Instituto de Ciências Biomédicas Abel Salazar, University Porto, Porto, Portugal; 4 Department of Neuroscience, Imaging and Clinical Sciences, School of Medicine and Health Sciences, “G. d’Annunzio” University of Chieti-Pescara, Chieti, Italy; 5 Unit of Molecular Genetics, Center for Advanced Studies and Technology (CAST), “G. d’Annunzio” University of Chieti-Pescara, Chieti, Italy

**Keywords:** multiple sclerosis, TNFα, miR-130a-3p, DNA methylation, saliva biomarkers, epigenetic regulation

## Abstract

**Background:**

Tumor necrosis factor alpha (TNFα) is a key cytokine involved in the inflammatory and neurodegenerative processes underlying multiple sclerosis (MS). Its expression is finely regulated by epigenetic and post-transcriptional mechanisms, including promoter methylation and miRNA activity. The objective of this study is to investigate TNFα expression, promoter methylation, and its regulation by miR-130a-3p in patients with relapsing–remitting (RR)MS, evaluating both serum and saliva as potential diagnostic biofluids.

**Methods:**

RRMS patients in clinical remission and sex and age-matched healthy controls (HC) were enrolled. TNFα levels were quantified in serum, peripheral blood mononuclear cell (PBMC) supernatants, and saliva using ELISA. TNFα mRNA expression and promoter methylation were analyzed by qPCR and pyrosequencing, respectively. Bioinformatic tools (TargetScan, miRTargetLink 2.0, miEAA 2) were used to explore miRNA–TNFα interactions, and miR-130a-3p expression was evaluated in serum and saliva by qPCR.

**Results:**

RRMS patients showed significantly higher TNFα mRNA and protein levels compared to HC, paralleled by significant hypomethylation of the TNFα promoter in PBMCs. miR-130a-3p was markedly downregulated in both serum and saliva, exhibiting an inverse trend with TNFα expression. Salivary TNFα levels mirrored serum alterations, supporting the feasibility of saliva as a noninvasive biomarker source.

**Conclusion:**

The data indicate that TNFα upregulation in RRMS is associated with promoter hypomethylation and reduced miR-130a-3p expression, suggesting a coordinated epigenetic and post-transcriptional control of this cytokine. The parallel trends observed in saliva and serum highlight the potential use of salivary TNFα and miR-130a-3p as minimally invasive biomarkers for MS monitoring and early diagnosis.

## Introduction

Multiple sclerosis (MS) is an autoimmune and neurodegenerative disorder characterized by chronic inflammation of the central nervous system (CNS) and has a high prevalence worldwide, affecting approximately two million individuals. MS primarily impacts young adults, and women at a 2-fold higher rate than men.

The pathogenesis of MS is complex, and it is postulated that a combination of genetic, epigenetic, microbial, and environmental factors may predispose individuals to MS (Olsson et al., 2017). MS is characterized by immune cell infiltration into the CNS, leading to both local and systemic inflammation. This infiltration results in neuroinflammation, axonal damage, and destruction of myelin, the protective sheath surrounding nerve fibers. This leads to disrupted communication between the brain and the rest of the body, manifesting in multiple symptoms, including motor, sensory, and cognitive impairments. Histopathologically, MS sclerosing lesions or plaques occur throughout the CNS but are commonly found in the periventricular regions, optic nerves, brainstem, and the spinal cord. They are characterized by focal demyelination, gliosis, and axonal loss (Bagnato et al., 2024). Clinically, the most frequent phenotype of MS is relapsing-remitting MS (RRMS), distinguished by episodes of reversible neurological attacks followed by total or local recovery. Over time, in the majority patients diagnosed with RRMS, neurologic deficits become permanent, and secondary progressive (SP) MS develops. However, 10%–15% of MS cases follow a primary-progressive (PP) course, in which patients accumulate progressive disability from the onset with no apparent distinct relapses (Lublin et al., 2014). Current research on the pathophysiological changes that occur in MS has identified agents such as cytokines, chemokines, nitric oxide, reactive oxygen species, glutamate, and free radicals, as well as mechanisms contributing to the establishment and maintenance of MS associated inflammatory processes. Among the different cytokines found elevated in patients, a growing body of evidence has indicated that tumor necrosis factor alpha (TNFα) plays a pivotal role in the MS development (Kahl et al., 2002), inducing a chronic inflammatory demyelinating disorder, synaptic instability in the brain, and subsequent sensory and cognitive damage that correlates with the degree of disability in MS patients (Begum et al., 2013; McCoy and Tansey, 2008).

Many cell types produce TNFα. Primarily activated macrophages, effector CD4^+^ and CD8^+^ T cells, and B lymphocytes do so in the peripheral blood, and they regulate each other’s function through the secretion of cytokines, forming a complicated network. Microglia are the main TNFα source within the brain, especially when stimulated by proinflammatory signals; however, neurons and astrocytes also contribute significantly to TNFα production (Probert, 2015; Heir et al., 2024). TNFα exerts potent proinflammatory activity by activating two specific TNFα receptors (TNFRs): TNF receptor type-1 (TNFR1) and type-2 (TNFR2) (Ribeiro et al., 2019). However, characterizing the precise mechanisms underlying TNFα signaling in human MS has proved difficult, and thus studies of MS animal models, such as experimental autoimmune encephalomyelitis (EAE), have led to hypotheses regarding the actions of TNFα (Mehta et al., 2018; Sayed et al., 2010).

Although studies on DNA methylation in MS are relatively few, there is increasing evidence that epigenetic modifications may confer risk for MS, and promoter methylation in a number of genes is reported to be associated with the status of multiple diseases and with functions of the immune system (Sokratous et al., 2016; Celarain and Tomas-Roig, 2020), highlighting the usefulness of evaluating DNA methylation in gene promoters for disease prediction and treatment.

MicroRNAs (miRNAs) are non-protein-coding, single-stranded, 18–25 nucleotide RNA molecules that do not encode any proteins but modify post-transcriptional gene expression, and multiple microRNAs can regulate the same mRNA sequence. Micro-RNAs have been recognized as potent regulators of several pathways and genes implicated in the pathophysiology of inflammatory disorders. Recent studies have found that miRNAs affecting glial cells and peripheral immune cells are involved in MS pathogenesis (Gao et al., 2021; Al-Temaimi et al., 2024; Gandhi, 2015). Given the hypothesis that measurement of the mRNA level of TNFα in peripheral blood mononuclear cells (PBMCs) and the protein level in serum could reflect the general TNFα expression status and the inter-regulation among cells, it is also necessary to consider epigenetic changes, such as DNA methylation and miRNA expression, which have been functionally linked to transcriptional and post-transcriptional modulation of associated genes in playing a significant role in regulating TNFα production and function.

Exploring the connection between TNFα, its miRNA targets, and promoter methylation in a noninvasive, safe, and easily accessible collection fluid may lay the foundation for identifying biomarkers to enable assessment of disease risk at the earliest possible stage, aid in the prevention of disease progression, and enhance public health outcomes by facilitating timely interventions. Towards this objective, our aim is to bridge the gap between potential salivary markers and those present in other biological fluids, such as cerebrospinal fluid (CSF) and serum, with a particular focus on TNFα, which could represent a clinically valuable diagnostic tool.

To address this issue, the expression, production, and circulating levels of TNFα, a gene-associated miRNA selected for its reported involvement in inflammation, as well as the promoter methylation of the TNFα gene, were evaluated in samples from patients with MS and in HC.

## Materials and methods

### Study population and ethical statement

The study included 14 subjects (7 males, 7 females) diagnosed with MS according to the McDonald 2010 criteria (Polman et al., 2011) and recruited at the Multiple Sclerosis Center of the University “G. d’Annunzio” of Chieti. Patient demographics (sex and age) and clinical data (disease course, disease duration, and disability level), evaluated using the Expanded Disability Status Scale (EDSS) (Kurtzke, 1983), were collected. All patients with MS were in the remission clinical phase, defined as the period of recovery with no relapse episodes and without any immunomodulatory treatment within the last 3 months before their time of enrollment in the study. They, therefore, were defined as having RRMS. The control group comprised 14 healthy subjects with similar demographic characteristics. Specifically, this control group did not present with clinical symptoms or laboratory parameters of inflammation, MS, or any other autoimmune disease. The local ethics committee (University “G. d’Annunzio” of Chieti) approved the study (CRRM; 2023; 07; 11; 03), and all participants signed informed written consent that followed the ethical standards laid down in the 1964 Declaration of Helsinki.

### Sample collection

A total of 20 mL of peripheral blood samples and 1 mL of serum were collected, respectively, in an EDTA tube and clot activator tube, from each RRMS subject and HC at the time of enrollment. Saliva sampling was performed for all participants between 9 a.m. and 11 a.m., by passive unstimulated drooling into a sterile tube. Specimens were processed within 1–2 h of collection. Serum and saliva were obtained by centrifugation (1500xg for 10 min) and aliquoted into tubes for storage at −80 °C until use.

PBMCs were isolated from EDTA tubes by centrifugation using a Ficoll-Hypaque density gradient. Cells were harvested from the interface, washed twice with Dulbecco’s phosphate-buffered saline (DPBS), and resuspended in RPMI 1640 medium (Merck KGaA, Germany) supplemented with 10% fetal bovine serum (FBS) (Euroclone S. p.a, Italy), 4 mM L-glutamine, 50 U/mL penicillin, and 50 mg/L streptomycin (Merck KGaA, Germany). Cells were then seeded at a concentration of 2 × 10^6^ in 5 mL polypropylene culture tubes (BD Falcon™, Two Oak Park Bedford, United States) and grown in an incubator at 37 °C and 5% CO_2_ for 24 h.

All reagents were screened before use and found to be negative for endotoxin (<10 pg/mL; Associates of Cape Cod, Inc., Woods Hole, MA, United States) and *mycoplasma* contamination (MycoAlert™ *Mycoplasma* Detection Kits, Lonza Bioscience, United States). More than 98% of the PBMCs were viable, as determined by trypan blue dye exclusion at the beginning of the culture, and more than 90% were viable before the supernatants were collected.

### TNFα enzyme-linked immunosorbent assay

TNFα concentrations in serum, PBMC culture supernatants, and saliva were evaluated using an enzyme-linked immunosorbent assay (ELISA) with a commercially available “Human TNFα ELISA Kit” (OriGene Technologies GmbH, United States) with a specificity of 70 ng/mL and a sensitivity of 7 pg/mL. To ensure the accurate performance of the immunoassay in the salivary matrix, representative samples of saliva were first tested for dilution and recovery. The optimal dilution of saliva was determined to ensure that measurements were performed in the dynamic range of the assay. All samples were analyzed in duplicate at the same time and reading, at 450 nm λ, of the relative concentrations were performed using the Glomax Multidetection System (Promega, Italy).

### RNA extraction and real-time polymerase chain reaction

Total RNA was extracted from PBMCs using QIAzol reagent (Qiagen, Germany) according to the manufacturer’s protocol. The RNA concentration was determined by measuring the absorbance of the samples at 260 nm λ using a NanoDrop 2000 UV-Vis Spectrophotometer (Thermo Scientific, United States), and its purity was assessed by the absorbance ratio of 260/280 nm and 260/230 nm λ (Fleige and Pfaffl, 2006). For each sample, 1 μg of RNA was reverse transcribed into complementary DNA (cDNA) using the QuantiTect Reverse Transcription Kit (Qiagen, Germany). Subsequently, Real-Time Polymerase Chain Reaction (RT-PCR) was performed using the GoTaq® qPCR Master Mix (Promega, Italy), to evaluate the gene expression of TNFα and Ribosomal Protein S18 (RPS18). Specific primer pairs were used to evaluate the gene expression of TNFα (FW:5′-CCTTCCTGATCGTGGCAG-3’; RW:5′-GCTTGAGGGTTTGCTACAAC-3′), and the housekeeping gene RPS18 (FW 5′-CTTTGCCATCACTGCCATTAAG-3′, RW 5′-TCCATCCTTTACATCCTTCTGTC-3′).

The expression of the TNFα gene was first normalized with the expression of the housekeeping gene, obtaining a ΔCt for patients and controls (ΔCt = Ct_TNFα_–Ct_18s_). The ΔΔCt was then calculated (ΔΔCt = ΔCt patient–ΔCt control), and changes in gene expression levels were expressed as 2^−ΔΔCt^ (Livak and Schmittgen, 2001). All RT-PCR reactions were performed in triplicate using a CFX96 Real-Time PCR Detection System (Biorad, United States) under the following conditions: initial incubation at 95 °C for 2 min, followed by 40 cycles at 95 °C for 30 s, followed by 60 °C for 1 min and 30 s at 68 °C.

### DNA isolation and pyrosequencing

Genomic DNA was extracted from blood samples using the NucleoSpin Tissue Kit (Macherey-Nagel, Germany). The quantity and quality of the DNA were assessed using Qubit 2.0 (Invitrogen, Italy). DNA was bisulfite-converted using the EpiTect Plus DNA Bisulfite Kit (Qiagen, Germany), converting unmethylated cytosines to uracils, and stored at −20 °C until use. PCR amplification of the TNFα promoters was performed using 2× PyroMark PCR Master Mix and 10× Coral Load Concentrate (Qiagen, Germany), 0.2 μM of each primer, 1 μL of bisulfite-converted DNA, and nuclease-free water to a final volume of 25 μL. The sequences of primers used for DNA methylation analysis are: FW PCR primer 5’-[Biotin]- TGAGGGGTATTTTTGATGTTTGT-3′; RW PCR Primer 5′-CCAACAACT ACCTTTATATATCCC-3′; Sequencing Primer 5′-ATAAACCCTACACCTTCTAT-3′ and Sequence to Analyze CTCA/GATTTCTTCTCCATCA/GCA/GAAAACA/GAAAATTT.

The PCR conditions were as follows: 57 °C for 30s, 72 °C for 30 s, and a final extension at 72 °C for 10 min. To evaluate the methylation status of specific CpG sites of major interest located in the promoters of the TNFα gene in a single experimental approach, the pyrosequencing reaction plates were loaded on a PyroMark Q96ID (Qiagen, Germany), and CpG methylation analysis was conducted using PyroMark CpG software (Qiagen, Germany). Triplicates were generated for each PCR. The methylation of each amplicon was calculated as the median methylation status of each analyzed CpG. Four CpGs were evaluated in the TNFα gene promoter.

### Identification and analysis of miRNA

Saliva and serum samples from RRMS patients and HC were used to isolate miRNAs using the miRNeasy kit (Qiagen, Germany), following the manufacturer’s instructions. This approach ensured the extraction of miRNAs of high purity and quality, which is essential for the accuracy of subsequent molecular analyses. The concentration and quality of the extracted miRNAs were determined by spectrophotometry using NanoDrop 2000UV-Vis (Thermo Fisher, United States) at λ = 260 nm. For complementary DNA (cDNA) synthesis, miRNAs were reverse transcribed using the miRCURY LNA RT kit (Qiagen, Germany). Reverse transcription was performed using 5 ng of total RNA for each sample, following the manufacturer’s protocol, to obtain high-quality cDNA. The analysis of miR-130a-3p, identified through bioinformatics and literature analysis, was performed using miR-16 as a housekeeping gene. miRNA expression levels in the serum and saliva samples of RRMS patients and HC were evaluated using Real-time PCR with the CFX96 Real-Time PCR Detection System (Bio-Rad, Hercules, CA, United States). Gene expression analysis was performed using the 2^−ΔΔCt^ method.

### Bioinformatics analysis of TNF targets

Bioinformatic analysis using the prediction software TargetScan Human (https://www.targetscan.org), miRTargetLink 2.0 (https://ccb-compute.cs.uni-saarland.de/mirtargetlink2) and miEAA 2 (https://ccb-compute2.cs.uni-saarland.de/mieaa) was performed to identify key miRNAs involved through the TNF-α-activated signaling pathway in MS progression. TargetScan predicts microRNA (miRNA)–mRNA interactions by identifying conserved complementarity between the miRNA seed region and the 3′untranslated regions (3′-UTRs) of target genes, integrating evolutionary conservation and context scoring to prioritize predictions. miRTargetLink 2.0 is an interactive platform for the visualization and exploration of both experimentally validated and predicted miRNA–target relationships, enabling network-based analysis and functional filtering, and miEAA 2 (microRNA Enrichment Analysis and Annotation tool, version 2) performs enrichment and overrepresentation analyses on defined miRNA sets, integrating multiple annotation databases to identify significantly enriched pathways, biological functions, and disease associations.

### Statistical analysis

The anamnestic and clinical data of the patients were summarized as quantitative variables and reported as median and interquartile range (IQR), and qualitative variables as frequencies and percentages. The statistical differences in gene expression fold changes between MS patients and HC subjects were determined using a 95% confidence interval. The Mann-Whitney U test was used to compare quantitative variables between the two groups. Comparative analysis and correlation were evaluated using Spearman (rho). In all tests, the threshold for statistical significance was set at p < 0.05. Data were analyzed using GraphPad software.

## Results

### Patient demographics, clinical characteristics, and TNFα serum levels

The study group comprised 14 patients with RRMS (seven women and seven men; median age, 45.0 years; range, 36–54 years) and 14 HC (eight Women and six men; median age, 46 years; range, 37–57 years). There were no significant differences in age and sex ratio between the patients and controls (p > 0.05). The median disease duration was 15 years (range 5–21). The median EDSS score in patients with RRMS was 3.7 (range 2–5.8) (Table 1).

**TABLE 1:** Features of patients with RRMS and HC.

Variables		MS (n = 14)	HC (14)
Sex, n (%)			
	Male	7 (50%)	6 (42.85%)
	Female	7 (50%)	8 (57.14%)
Age (years), mean (DS)		45 (±9)	46 (±9)
Smoke, n (%)			
	No	8 (57.14%)	5 (35.71%)
	Yes	4 (28.57%)	3 (21.42%)
	Ex	2 (14.28%)	6 (42.85%)
EDSS, median (IQR)		3.7 (2.0–5.8)	—
Age of onset (years), median (IQR)		31 (23–35)	—
Disease duration (years), median (IQR)		15 (5–21)	—
OCB, n (%)			—
	Present	8 (57.14%)	—
	Absent	1 (7.14%)	—
	nd	5 (35.71%)	—

nd: non detected.

**FIGURE 1:**
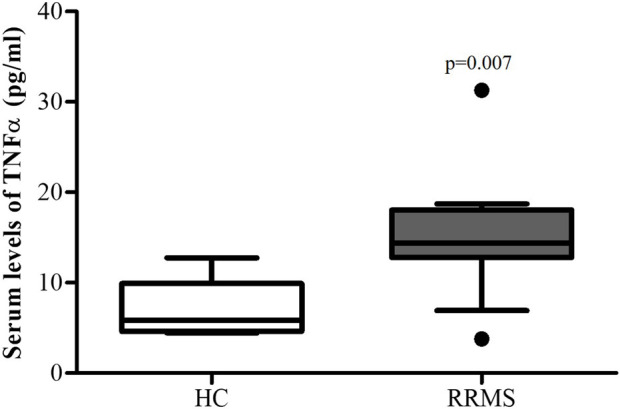
Serum TNFα levels in patients with MS and HC subjects. Data are reported as median ±standard deviation (SD). MS vs. healthy subjects p < 0.05.

Serum levels of TNFα in patients with RRMS were significantly (p = 0.007) higher than those in the control group (14.4 ± 6.5 pg/mL vs. 5.8 ± 3.2 pg/mL, respectively) (Figure 1). There was no statistically significant correlation between the serum TNFα level and EDSS score in RRMS patients.

### Expression and production of TNFα in PBMCs

**FIGURE 2:**
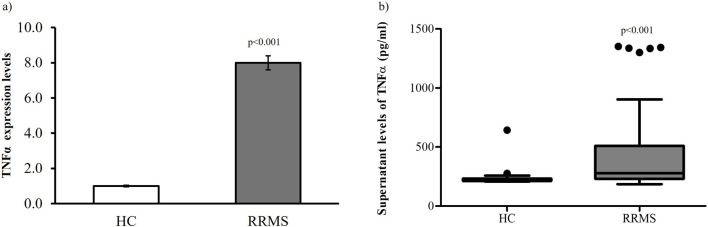
**(a)** TNFα expression levels in PBMCs isolated from RRMS patients and HC subjects. Data are reported as median ±standard deviation. RRMS vs. HC p < 0.05. **(b)** Cell-free supernatant TNFα levels in RRMS patients and HC subjects. Data are reported as median ±standard deviation. MS vs. HC p < 0.05.

TNFα serum levels represent the sum of immune and non-immune cellular sources and are influenced by the clearance rate. To determine whether the higher circulating levels observed in RRMS patients result from increased production and gene transduction by PBMCs, we evaluated TNFα expression and production in PBMCs isolated from both MS patients and HC subjects (Figure 2a). In RRMS patients, the expression levels of TNFα were significantly higher than those in HC subjects, in accordance with the levels of TNFα detected in the supernatant of cell culture (Figure 2b).

### TNFα epigenetic regulation

#### Methylation analysis

Changes in promoter methylation of the TNFα gene and their effects on gene expression were analyzed. Total methylation, as the mean value of all CpG sites analyzed, comparing 14 HC with 14 RRMS samples, revealed a significant reduction in CpG methylation in its promoter region (p = 0.001).

This finding implicates the involvement of promoter methylation in the regulation of TNFα expression, the significant role of TNFα methylation in the immune system, and its potential as an epigenetic marker for diseases. At the single CpG level, the methylation values of CpG2, CpG3, and CpG4 in PBMCs showed significant hypomethylation, supporting the notion that the upregulation of TNFα protein levels in PBMCs is probably related to differential DNA methylation.

**FIGURE 3:**
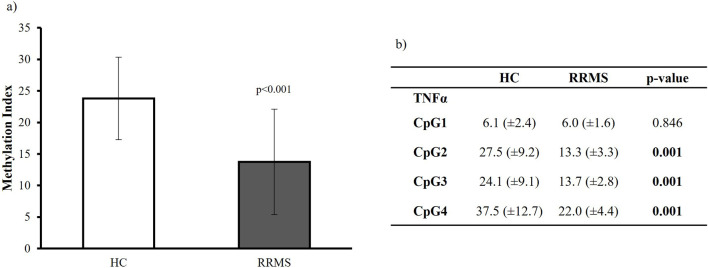
**(a)** TNFα methylation index in patients with MS and HC subjects. Data are reported as median ±standard deviation. MS vs. HC subjects p < 0.05. **(b)** TNFα methylation levels of individual CpG dinucleotides.

We detected a negative association between TNFα promoter methylation and gene expression in PBMCs, and an association analysis was conducted by evaluating changes in promoter methylation and gene expression (Figure 3).

TNFα promoter methylation in the blood was not correlated with sex or age (data not shown).

#### miRNA evaluation

Gene expression is not only regulated by genetic sequence methylation but is also influenced by other epigenetic mechanisms, including non-coding RNAs, such as miRNAs.

To explore the relevance between TNFα and miRNA, we applied the bioinformatic prediction software TargetScan Human (https://www.targetscan.org) and miRTargetLink 2.0 (https://ccb-compute.cs.uni-saarland.de/mirtargetlink2). The main target gene predicted by TargetScan Human was miR-130a-3p, as reported in Table 2, with the complementary sequence identified in the 3′-UTR of TNFα, followed by other miRNAs with less site specificity.

**TABLE 2:** TargetScan Human predicted complementary sequence of TNFα.

	Predicted miRNA	Site type	Context++ score	Context++ score percentile
hsa-miR-130a-3p	5' …UCCCUCUAUUUAUGUUUGCACUU… |||| ||||||| 3′UACGGGAAAAUUGUAACGUGAC	7mer-m8	−0.38	98
hsa-miR-454-3p	5'…UCCCUCUAUUUAUGUUUGCACUU… ||||||| 3′UGGGAUAUUCGUUAUAACGUGAU	7mer-m8	−0.36	98
hsa-miR-3666	5'…UCCCUCUAUUUAUGUUUGCACUU… ||||||| 3′AGCCGUAGAUGUGAACGUGAC	7mer-m8	−0.35	98
hsa-miR-301a-3p	5'…UCCCUCUAUUUAUGUUUGCACUU… ||||||| 3′CGAAACUGUUAUGAUAACGUGAC	7mer-m8	−0.34	98
hsa-miR-130b-3p	5'…UCCCUCUAUUUAUGU-UUGCACUU… ||| ||||||| 3′UACGGGAAAGUAGUAACGUGAC	7mer-m8	−0.36	98
hsa-miR-301b-3p	5'…UCCCUCUAUUUAUGUUUGCACUU… ||||||| 3′CGAAACUGUUAUAGUAACGUGAC	7mer-m8	−0.34	97
hsa-miR-4295	5'…UCCCUCUAUUUAUGUUUGCACUU… ||||||| 3′UUCCUUUUGUAACGUGAC	7mer-m8	−0.30	96

Context++ score and features that contribute to the Context ++ score percentile were evaluated as in Agarwal et al. (2015).

**FIGURE 4:**
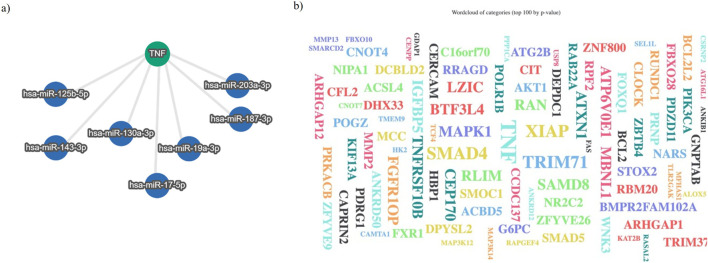
**(a)** Interaction graph obtained using miRTargetLink 2.0; **(b)** Visualization of a word-cloud of the target for identified miRNAs, with relevance for TNFα, associated with the seven searched miRNAs.

Moreover, analysis with miRTargetLink 2.0 identified miR-130a-3p as a strongly validated target of TNFα (Figure 4a), supporting its potential role in TNFα-mediated signaling. To further explore the functional context, a complementary enrichment analysis was conducted using miEAA 2 (https://ccb-compute2.cs.uni-saarland.de/mieaa). This analysis revealed a significant overrepresentation of TNFα as the principal subcategory, reflecting the observed distribution of counts across the identified miRNAs. The results are visually summarized in a word cloud plot, where the prominence of TNFα underscores its central involvement in the regulatory network (Figure 4b; Table 3).

**TABLE 3:** Interaction table obtained using miRTargetLink 2.0

miRNA	Target/Target pathway	Support	Source	Reference - PMID
hsa-miR-130a-3p	TNFα	Functional MTI	MIRT054762	24885472/26435691
hsa-miR-125b-5p	TNFα	Functional MTI	MIRT733472	26435691
hsa-miR-143-3p	TNFα	Functional MTI	MIRT437418	23804075
hsa-miR-17-5p	TNFα	Functional MTI	MIRT734730	26041742
hsa-miR-187-3p	TNFα	Functional MTI	MIRT054325	23071313
hsa-miR-19a-3p	TNFα	Functional MTI	MIRT006787	21271217/26041742
hsa-miR-203a-3p	TNFα	Functional MTI	MIRT006857	22917968

**FIGURE 5:**
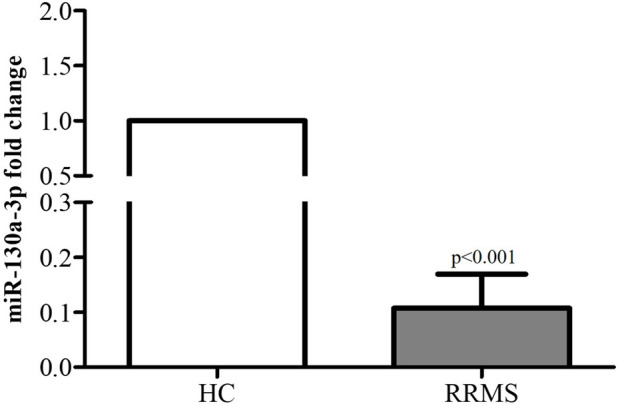
miR-130a-3p levels in RRMS patients and HC subjects. Data are reported as median ±standard deviation. RRMS vs. HC subjects, p < 0.001.

Moreover, we assessed miR-130a-3p expression levels in patients with RRMS. Interestingly, real-time PCR analysis revealed that miR-130a-3p was significantly downregulated in the serum of RRMS patients as compared to that in HC subjects (Figure 5). Based on the results, an inverse correlation emerged, suggesting that the reduced levels of this miRNA relieve its repression on TNFα translation, thereby contributing to its increased gene expression and production levels.

#### Comparative evaluation of serum and salivary samples

Considering the known relationship between serum and saliva, both of which represent biological systems useful in the evaluation of key molecules indicative of the systemic health status of an individual, we evaluated whether this correlation was present in our patients with RRMS in relation to TNFα and miR-130a-3p.

**FIGURE 6:**
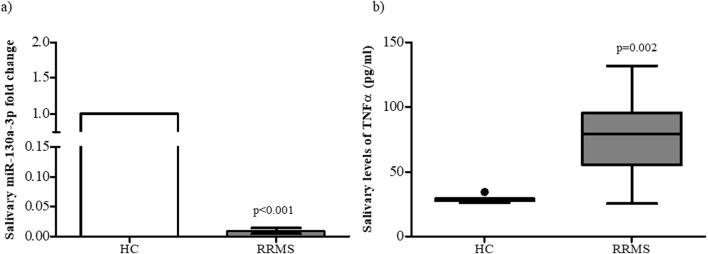
**(a)** miR-130a-3p gene expression levels in RRMS patients compared to HC subjects. Data are reported as median ±standard deviation. RRMS vs. HC subjects p < 0.001. **(b)** TNFα levels in the saliva of RRMS patients and HC subjects. Data are reported as median ±standard deviation. MS vs. HC p < 0.05.

First, we evaluated the levels of TNFα in saliva using ELISA and detected a significant increase in RRMS patients compared to HC subjects (p = 0.002), in agreement with the increase in serum levels. Interestingly, TNFα levels detected in saliva were higher than those measured in serum (66.6 ± 23.03 pg/mL and 14.13 ± 7 pg/mL, respectively), indicating intense endogenous production (Ferrari et al., 2023) (Figure 6a).

Subsequently, miR-130a-3p gene expression levels were assessed in saliva samples, and a significant reduction was observed (p < 0.001), following the well-established biological principle that miRNA expression inversely regulates the production of their target proteins (Figure 6b). Spearman correlation analysis showed a negative correlation between TNFα production and miR130a-3p expression in saliva, although this was not statistically significant (r = −0.13; p = 0.71).

## Discussion

TNFα plays a crucial role in immunomodulation and inflammatory responses (Bradley, 2008), highlighting the importance of elucidating the mechanisms regulating its expression. In this light, several studies have demonstrated the involvement of TNFα signaling in the pathogenesis of MS, and its correlation with disease severity (Sharief and Hentges, 1991; Fresegna et al., 2020). Whereas much of our knowledge derives from studies using the rodent EAE model of MS, the exact mechanisms underlying TNFα signaling in patients with MS have remained difficult to dissect. Nevertheless, TNFα appears to contribute to immune cell activation and infiltration into the CNS, as well as to the ensuing demyelination and axonal injury (Mazziotti et al., 2024).

Peripheral TNFα signaling increases blood–brain barrier (BBB) permeability and promotes immune cell activation and trafficking, while elevated TNFα levels within the CNS further enhance microglial and astrocytic activation and antibody and cytokine secretion, ultimately leading to oligodendrocyte death, demyelination, and neuronal damage (Hofman et al., 1989). In addition, TNFα has been implicated in synaptic dysfunction; peripheral TNFα production during preclinical EAE has been shown to induce synaptic instability in the primary somatosensory cortex, potentially contributing to sensory and cognitive impairments (Yang et al., 2013).

Of note, TNFα is reported to exert both pathogenic and protective effects in MS, depending on the receptor subtype engagement: signaling via TNFR1 generally promotes inflammation, apoptosis, and demyelination, whereas TNFR2 activation supports remyelination and neuroprotection, particularly through regulatory T cells and oligodendrocyte precursor cells (Arnett et al., 2001; Fresegna et al., 2020). This dual role complicates therapeutic approaches targeting TNFα, as illustrated by the paradoxical worsening of MS observed in patients treated with nonselective TNFα inhibitors (Kemanetzoglou and Andreadou 2017).

At the molecular level, TNFα expressions are tightly regulated at both the transcriptional and post-transcriptional levels (Falvo et al., 2010). Epigenetic mechanisms play a central role in regulating this process. DNA methylation has been shown to inversely correlate with TNFα transcription, and aberrant methylation patterns have been reported in MS patients during relapse. Moreover, histone modifications influence the accessibility of TNFα promoter regions, contributing to disease-specific expression patterns.

MicroRNAs add a layer of regulation by post-transcriptionally controlling TNFα and related signaling pathways. For example, dysregulation of miR-146a and miR-155, both of which are key modulators of innate and adaptive immune responses, has been associated with MS pathogenesis and progression (Koch et al., 2013; Junker et al., 2009). MiR-146a generally exerts anti-inflammatory effects, whereas miR-155 promotes pro-inflammatory responses, highlighting the complex interplay of non-coding RNAs in shaping TNFα-mediated pathologies.

Collectively, these findings emphasize that TNFα is a pivotal mediator in MS, acting at multiple levels of disease initiation and progression, with its expression and signaling tightly modulated by epigenetic and posttranscriptional mechanisms. A deeper understanding of these regulatory networks could open new therapeutic opportunities, particularly strategies targeting epigenetic regulators and miRNAs to fine-tune TNFα expression in a cell-type- and context-specific manner.

In the current study, first we detected higher TNFα mRNA and protein levels in patients with RRMS than in HC subjects, and this finding is consistent with other reports in the field (Mazziotti et al., 2024). To test the hypothesis that the observed increased TNFα levels in the serum of RRMS patients is triggered by upregulation of mRNA expression in PBMCs, the spontaneous expression of TNFα was evaluated, and significantly higher expression was detected in RRMS patients than in HC subjects, in accordance with the levels of TNFα detected in cell-free supernatants. Our deliberate focus on treatment-free RRMS patients in clinical remission was aimed to isolate disease-related molecular signals by reducing two major confounding sources: relapse-driven inflammation and therapy-induced epigenetic modulation. Relapses and remission differ meaningfully at the level of circulating small RNAs, with several studies showing opposite or state-dependent shifts in miRNA profiles, which could mask or mimic associations in the event that activity phases are mixed within a small cohort (Muñoz-Culla et al., 2016). Likewise, multiple disease-modifying therapies may potentially reshape epigenetic and post-transcriptional landscapes, for example, interferon-β altering DNA-methylation at interferon-response genes, fumarates affecting miR-21 methylation, and natalizumab normalizing or modulating dysregulated miRNAs; therefore, excluding recently treated patients was necessary to avoid pharmacologic signature overlay (Xavier et al. 2023). However, this design choice narrows generalizability. Progressive phenotypes exhibit distinct molecular architectures, including genetic–epigenetic and transcriptomic features in PPMS, and biomarker differences relative to RRMS; therefore, our hypomethylation and miRNA findings should not be assumed to be representative of SPMS, PPMS, or relapse states without direct testing (Pahlevan Kakhki et al., 2024). Additional proteomic screening studies and evaluation of TNFα and miR-130a-3p expression levels in a larger population are necessary to identify biomarkers and post-translational modifications to understand their ability to differentiate whether patients are in the RRMS course or have converted to the SPMS course or are in the impending flare-up of MS.

Recent reviews have also underscored that MS epigenetics and miRNA biology are heterogeneous across stages and compartments, reinforcing the need for validation in broader phenotypes and during clinically active phases (Manna et al., 2024). Subsequently, we tested the hypothesis that in RRMS patients, hypomethylation of the TNFα promoter region upregulates the expression of this gene. Our results showed that at the level of individual CpG islands, the methylation values of CpGs 2–4 were significantly hypomethylated. This suggests that the hypomethylation status of the TNFα promoter region in RRMS may be a determinant of increased TNFα mRNA expression. The inverse relationship between TNFα promoter methylation and mRNA expression broadens the understanding of the regulatory mechanisms involved in TNFα expression in RRMS and suggests the potential application of TNFα promoter methylation as an epigenetic marker of RRMS pathogenesis.

Several studies have shown that the expression of several miRNAs involved in innate immunity and inflammation is altered in PBMCs, CSF, and brain tissue of MS patients (Gao et al., 2021; Ridolfi et al., 2013). Notably, miRNAs are detectable across body fluids (e.g., plasma, serum, CSF, and urine), and their remarkable stability in extracellular environments makes them attractive candidates for minimally invasive biomarkers. Specific circulating miRNA signatures have been proposed as potential diagnostic and prognostic biomarkers for RRMS, with some profiles correlating with disease activity and treatment responses (Jagot and Davoust, 2016; Muñoz-San Martín et al., 2022). Functionally, miRNAs are not merely passive biomarkers; they actively regulate pathogenic processes. For example, miR-155 and miR-326 promote Th1/Th17 inflammatory responses, whereas miR-146a negatively modulates innate immunity (Wu et al., 2016). Alterations in miRNA expression in oligodendrocytes and neurons can influence demyelination, remyelination, and synaptic integrity, directly linking them to disease progression. These mechanisms suggest that miRNAs may serve not only as biomarkers for disease diagnosis and monitoring but also as potential therapeutic targets, offering new perspectives for precision medicine in MS.

In this regard, miR-130a-3p has attracted attention in clinical settings. A serum study found that miR-130a-3p, along with miR-221-3p and miR-146a-3p, exhibited high accuracy in stratifying patient response, suggesting a potential role as a biomarker (Wade et al., 2020). Furthermore, a circulating prognostic score (in oncology), based on combined expression, including Exo-miR-130a-3p, demonstrated predictive capabilities in disease progression (Marconi et al., 2022). Although not specific to MS, these data underscore the potential value of miR-130a-3p as a biomarker in inflammatory and degenerative diseases.

Recently, MiRNA-130a has been found to be implicated in critical processes across various human diseases and miR-130a has been reported to directly target and negatively regulate TNFα expression. Since the involvement of miRNA-130a-3p in the regulation of inflammation during the development of MS has not been fully clarified and remains poorly understood, we evaluated circulating levels of both miRNA130a and TNFα, and obtained the following data. First, increased TNFα and decreased miRNA-130a were observed in serum as well in saliva of MS patients, suggesting that epigenetic regulation of TNFα was mediated, at least partially, through downregulation of miRNA-130a and TNFα promoter methylation upregulation of TNFα. A second interesting result was obtained by evaluating levels of TNFα and miRNA-130a-3p in saliva from RRMS patients and HC subjects, and highlighted comparable trends to those observed in serum. In this regard, saliva represents a promising diagnostic biofluid whose collection is noninvasive and stress-free. Indeed, large quantities can be obtained without need of special storage conditions or clinician training (Goldoni et al., 2022). Moreover, saliva is easier to handle than blood, it does not clot, it is stable over time and provides little to no risk of cross-contamination or exposure to pathogens, particularly in comparison to CSF sampling that generally involves a highly invasive procedure and hospitalization (Cross et al., 2024). In addition, the high comparative cost for the procedure and patient compliance make it difficult to perform repeated measurements involving CSF.

Some suggestions have been proposed to explain the presence of TNFα and miRNA in saliva. These include the passage from the blood via active transport, passive intracellular diffusion, microfiltration, production by the salivary gland-infiltrating T helper 1 and T cytotoxic 1 cells and salivary gland epithelium (Gleerup et al., 2019; Reale et al., 2020; Costantini et al.2020).

In the present study, low expression of miR-130a-3p was observed in the RRMS patients. Consistently, the dysregulation of miR-130a-3p has also been identified in other neurodegenerative diseases. In Parkinson’s disease, low expression of miR-130a-3p was detected, and overexpression of miR-130a-3p is involved in the neuroprotective role of laminin-511 (LM511)-Yes-associated protein 1 (YAP) signaling against neuron degeneration during Parkinson’s disease (Zhang et al., 2017). In addition, in a study describing the beneficial effect of intermittent fasting in Alzheimer’s disease, miR-130a-3p was suggested to be involved in protective mechanisms (Greco and Rameshwar, 2007). These data support our hypothesis that miR-130a-3p and its decline are implicated in the development of RRMS.

In our RRMS remission cohort, we did not observe a significant correlation between serum TNFα and EDSS, nor between TNFα and salivary miR-130a-3p (inverse trend, r = −0.13; p = 0.71). Two non-exclusive explanations merit consideration. First, statistical considerations: the small sample and relatively narrow EDSS range likely reduced power to detect small-to-moderate associations, increasing the risk of a type II error; thus, the null findings should be interpreted as inconclusive rather than as evidence of no relationship (Serdar et al., 2021). Second, biological considerations: during clinical remission, peripheral TNFα can reflect transient immune activity and systemic influences, such as circadian variation, rather than the cumulative neurological disability indexed by EDSS, which accrues over years through progression independent of relapses. This temporal and compartmental mismatch can weaken cross-sectional correlations (Zielinski and Gibbons, 2022). Matrix specificity may further contribute, because cytokine–miRNA relationships differ across biological fluids (serum vs. saliva vs. CNS), and saliva, in particular, is sensitive to local oral inflammatory status and pre-analytical factors (e.g., collection timing, fasting, handling), which can add variability to a small patient number (Song et al., 2023).

Regarding saliva, the inverse but non-significant TNFα–miR-130a-3p trend suggests a plausible directionality. However, the effect size appears modest with wide uncertainty; replication in a larger, prospectively powered cohort - ideally with standardized sampling across times of day, is warranted (Lakens, 2022).

The study of Ruiz-Algueró et al. suggested that MS may begin earlier than previously thought (Ruiz-Algueró et al., 2025). Thus, in addition to the evaluation of potential risk factors such as exposure to Epstein-Barr virus (EBV) or genetic susceptibility, the early evaluation of potential biomarkers may help to recognize and manage MS earlier in its disease course. Our findings suggesting that the pathogenic mechanisms of RRMS may be mediated, at least in part, by the downregulation of miR-130a-3p and the upregulation of TNFα can pave the way for additional studies. In fact, although an association between TNFα and miR-130a-3p was observed, we could not establish a direct mutual regulatory relationship in RRMS. Nevertheless, the combined analysis of TNFα and miR-130a-3p level variations, including their detection in saliva, may represent a promising approach to support MS diagnosis. In this regard, a greater understanding of the complex molecular processes by which TNFα is involved in the pathogenesis and progression of MS requires further research. Specifically, by increasing the sample size, including subjects with different MS disease courses and tracing clinical parameters of the patients included in this study.

## Data Availability

The raw data supporting the conclusions of this article will be made available by the authors, without undue reservation.
